# The outweigh of toxicity versus risk of recurrence for adjuvant interferon therapy: a survey in German melanoma patients and their treating physicians

**DOI:** 10.18632/oncotarget.25439

**Published:** 2018-05-25

**Authors:** Katharina C. Kähler, Christine Blome, Andrea Forschner, Ralf Gutzmer, Axel Hauschild, Lucie Heinzerling, Elisabeth Livingstone, Carmen Loquai, Tina Müller-Brenne, Dirk Schadendorf, Jochen Utikal, Tobias Wagner, Matthias Augustin

**Affiliations:** ^1^ Department of Dermatology, Skin Cancer Center, University Hospital Schleswig-Holstein (UKSH), Campus Kiel, Kiel, Germany; ^2^ Institute for Health Services Research in Dermatology and Nursing (IVDP), University Medical Center Hamburg, Hamburg, Germany; ^3^ Department of Dermatology, Eberhard-Karls University of Tübingen, Tübingen, Germany; ^4^ Department of Dermatology, Skin Cancer Center Hannover, Hannover Medical School, Hannover, Germany; ^5^ Department of Dermatology, University Hospital Erlangen, Erlangen, Germany; ^6^ Department of Dermatology, University Hospital Essen, University Duisburg-Essen, Essen, Germany; ^7^ Department of Dermatology, University of Mainz, Mainz, Germany; ^8^ Department of Dermatology, Venereology and Allergology, University Medical Center Mannheim, Mannheim, Germany

**Keywords:** melanoma, patient preferences, interferon

## Abstract

After more than two decades with interferon alfa-2a and 2b (IFN) as the only approved drugs in the adjuvant setting for melanoma, new treatment approaches like immune checkpoint inhibitors and BRAF-MEK inhibitors improve the progression free survival (PFS) and also the overall survival (OS).

We compared physicians' preferences (“utilities”) for health states associated with IFN therapy to their patients' preferences. Utilities describe a preference for a specific health state on a scale of 0 (as bad as death) to 1.0 (perfect health).

**Setting:**

We assessed utilities for health states associated with adjuvant IFN using the standard gamble technique in 108 physicians and 130 melanoma patients. Four IFN toxicity scenarios and three outcome scenarios were given to the participants. Both groups were asked for the 5-year disease free survival (DFS) they would need to accept the described IFN-related side effects.

**Results:**

In both groups, utilities for melanoma relapse were significantly lower than for IFN side effects, showing that toxicity was more acceptable than relapse. Physicians indicated higher utilities for each scenario and needed lower 5-year DFS both in case of mild-to-moderate and severe side effects. Patients were willing to tolerate mild-to-moderate and severe toxicity for a 50% and 75% chance of 5-year DFS, while physicians only required a chance of 40% and 50%, respectively.

**Conclusion:**

Both physicians and patients rated melanoma recurrence much lower than even severe IFN side effects. In direct comparison, physicians rated cancer-related scenarios more positively and accepted IFN toxicity for an even lower treatment benefit.

## INTRODUCTION

The high risk melanoma (nodal involvement AJCC stage III) patient population is heterogeneous, with DSF rates of 78%, 59% and 40% for stage IIIA, IIIB and IIIC, respectively [1–3]. IFN has only limited activity for adjuvant treatment of melanoma (DFS HR.86) [4]. New, recently approved drug modalities like BRAF-MEK and immuncheckpoint inhibitors improved the prognosis of high risk-melanoma patients (DFS HR.47-.72) markedly [4, 5]. The concept of adjuvant treatment is to avoid subsequent distant metastasis and finally cancer death.

However, adjuvant treatment has a specific toxicity profile, which is why physicians need to thoroughly discuss the individual risk-benefit ratio with eligible patients. This decision making process may be influenced by the physician's personal convincement. Therefore, it is important to be aware of any differences between patients and physicians' attitudes towards toxicity. Although for more than twenty years, only IFN has been approved and available for high-risk patients [5], nothing is known about differences between melanoma patients' and their treating physicians' preferences towards IFN toxicity. Recently, clinical trials showed a positive effect of both targeted therapy and immune checkpoint blockade in the adjuvant setting [4, 6, 7]. With these new drug modalities, it is possible to improve DFS and for some also OS. So far, it is unknown how patients rate the risk-benefit ratio of these new drug modalities. We already know from other tumor entities that patients and physicians rate benefit-risk ratios differently [8, 9].

We evaluated the attitude towards IFN toxicity in patients and their treating physicians. Our aim was to evaluate which differences in terms of “trade-off” exist between melanoma patients and their treating physicians.

## RESULTS

119 physicians agreed to participate in the trial, of which 116 (drop out rate 2.5%) filled in the questionnaire and returned it to the melanoma center. The comparator group comprised 174 patients with a drop-out of 24 patients (drop out rate 13.8%). Three patients were excluded due to missing data.

Several plausibility checks testing for misordering of scenarios resulted in the exclusion of n=25 cases (9.5%) from analysis. The patient exclusion rate was higher (n=17, 11.6%) than the physician exclusion rate (n=8, 6.9%).

### Socio-demographic data (Table 1)

In the physician cohort, two thirds were female (n=116, 66.4%). We exclusively asked employed dermatologists; consequently, the highest age was 62 years. The youngest dermatologist aged 25 years. Mean age was 34.8 years ±7.1 SD. In the physician group, an equal graduation level could be presumed.

The mean duration of professional experience as a dermatologist was 6.9 years (±6.3 SD); the median was five years. The frequency of contact with melanoma patients per month had a mean of 65.6 and a median of 30 contacts. 92.2% of physicians indicated that they currently had contact with melanoma patients, 4.3% did not, and 3.4% did not answer the question. About half of the physicians (55.2%) were involved in the prescription of IFN therapy. 38.8% of physicians stated that they did not prescribe IFN. In the group of prescribing physicians (n=64), an average of 5.7 prescriptions were made per month with a wide range of 0.25 to 24 prescriptions per month (Table 2).

**Table 1:** Sociodemographics of the physician cohort in comparison to patient cohort

*Mean age* in years (SD, range)	Patients (n=130) 54.3 (±12.7, 25-82)		Physicians (n=108) 34.8 (±7.1, 25-62)	
	n	%	n	%
***Gender* (female)**	61	46.9	71	65.7
***Education level***				
· Low	20	15.4	0	0.0
· Intermediate	43	33.1	0	0.0
· High	64	49.2	108	100.0
***Professional qualification***				
· University or polytechnic degree	49	37.7	108	100.0
· Apprenticeship	74	56.9	0	0.0
***Marital status***				
· Married/partnership	113	82.5	76	70.4
· Widowed	5	3.6	0	0.0
· Divorced/separated	8	5.8	1	0.9
· Single	11	8.0	27	25.0
***Living alone***	18	13.1	30	27.8
***Employment status***				
· Employed	90	61.2	108	100.0
· Not working	56	38.1	0	0.0
***Other somatic disease***	37	28.5		^*^)
***Own malignancies in the past***	23	17.7	4	3.7
***Malignancies of closely related persons***	114	87.7	67	62.0

**Table 2:** Professional experience of the physician cohort

	Mean	SD	Median	Min	Max	95% CI	Valid (n)	Missing (n)	Total (n)
Duration of being a dermatologist (years)	6.9	6.3	5	0	29	5.7-8.1	113	3	116
Frequency of contact with melanoma patients per month	65.6	75.4	30	0	400	51.5-79.7	112	4	116
Prescription of IFNa-2b therapy (per month)	3.1	5.1	1	0	24	2.1-4.1	109	7	116
Prescription of IFNa-2b therapy (per month): only active prescribers	*5.3*	*5.7*	*3*	*0.25*	*24*	*3.9-6.7*	*64*	*0*	64^*^
*Percentage of subjects treated who have mild side effects.*	*62.6*	*27.7*	*70*	*0*	*100*	*55.8-69.4*	*62*	*2*	64^*^
*Percentage of subjects treated who have severe side effect.*	*15.3*	*13.1*	*10*	*0*	*60*	*12.0-18.7*	*62*	*2*	64^*^

We assumed that one important component influencing the participants' utilities might be self-experience with cancer or the affection of relatives by cancer. In the physician group, 57.8% were affected by cancer (partner 5.2%, close friends 10.3%, close relatives 47.4%), and only 5 physicians (4.3%) reported own current or precedent malignancies.

The percentage of subjects living alone was more than twice as high among physicians as among patients (30.2% versus 13.6%), which can be explained by the age differences.

The patient cohort (n=150) nearly equally consisted of female and male subjects (48.3% female versus 51.0% male). Age ranged from 25 to 82 years with a mean of 54.6 years ±12.6. About two thirds of patients were in active working life, the remaining patients were retired. Within the patients in active working life (n=90), the number of working hours per week ranged from 6 to 60 with a mean of 34.9 hours ±11.2 SD. 6.1% of patients reported that they were currently affected by another cancer and a further 17.0% reported antecedent malignancies. 68.8% of subjects had closely related persons affected by cancer (partner 8.8%, close friends 20.4%, close relatives 61.9%).

Significant differences between patient and physician group were found in the following variables: in the physician group, the proportion of female participants was higher (p.003). Physicians more frequently lived alone (p.003) and were single (p.003). Patients more often had self-experience with cancer (p.001), but were also more often confronted with cancer in their social sphere (<p.001).

### Utilities

The scenarios A to D illustrated the range of potential outcomes during and post-adjuvant IFN, Scenario E was relapse after IFN and scenario F recurrence without precedent IFN therapy (Table 3). A high proportion of 58.3% of physicians (n=63) and 42.3% of patients (n=55) had a treatment utility of 1.0 for scenario A (no side effects). Scenario B (mild-to-moderate side effects) showed lower average utilities, but above 0.9 for both groups. The gap between patient and physician answers grew to 6.4%-pts., while the median was nearly unchanged as compared to scenario A. 46 patients (35.4%) and 45 physicians (41.7%) had a utility of 1.0, which is interpreted as perfect health, despite these mild-to-moderate side effects.

**Table 3:** Utilities: comparison between physicians and patients (Utility for a disease-free health state was defined as U_0_=1.0.)

	Patients (n=130)			Physicians (n=108)			
Scenarios	Mean	SD	Median	Mean	SD	Median	p
Utility A: no side effects	0.94	0.14	0.99	0.99	0.03	1.00	<0.001
Utility B: mild-moderate side effects	0.90	0.18	0.99	0.97	0.07	1.00	0.023
Utility C: laboratory side effects	0.88	0.20	0.99	0.95	0.09	0.99	0.004
Utility D: severe side effects	0.81	0.25	0.90	0.91	0.15	0.98	0.001
Utility E: IFN, recurrence, cancer death	0.60	0.32	0.50	0.74	0.24	0.80	0.003
Utility F: recurrence, cancer death	0.60	0.31	0.50	0.75	0.25	0.80	0.001

In scenario C, patients showed a lower preference for the associated laboratory abnormalities (−2.6%-pts.), while the physicians' utilities stayed widely unchanged (−1.4%-pts. on average), enlarging the gap between the groups to 7.6%-pts. Despite of the lower mean utility of patients, the median was stable at 99.0% and thus identical to the median found in scenario A and B. A utility of 1.0 was indicated by 35 patients (26.9%) and 40 physicians (37.0%) (Table 3).

In scenario D, the patients' utilities dropped by 7.0%-pts. as compared to scenario C, while this decrease was not the same in physicians. The percentage of participants with a utility of 1.0 was 16.2% in the patient group and 20.4% in the physician group (Table 3).

Scenario E, the melanoma relapse showed the lowest utilities in both groups. However, 3.1% of patients still had a utility of 1.0 (but no physicians). Furthermore, for this scenario, the gap between the groups was highest with 14.5%-pts.

Scenario F was identical to scenario E, but without preceding IFN treatment, and resulted in similar utilities.

### Threshold questions and attitudinal questions

The threshold benefit for the **absolute chance** of being melanoma-free at 5 years after adjuvant IFN treatment with mild-to-moderate side effects was considerably higher for patients (59.6% ±20.6 SD) than for physicians (42.3% ±15.6 SD) and was characterized by high standard deviations (Table 4). Much higher chances of being melanoma-free at 5 years after adjuvant IFN treatment were required in case of severe side effects: for patients, the mean threshold benefit was at 69.8% (±22.5 SD) in contrast to 53.6% (±18.7 SD) in the physician group (Table 4).

**Table 4:** Threshold benefit and minimum risk reduction: comparison between physicians and patients: which chance (%) of being melanoma-free after 5 years is needed to accept toxicity

	Patients (n=130)			Physicians (n=108)			
Scenarios	Mean	SD	Median	Mean	SD	Median	p
**Threshold benefit: Chance of being melanoma-free at 5 years after adjuvant IFNa-2b treatment with mild-to-moderate side effects**	0.60	0.21	0.50	0.42	0.16	0.40	<0.001
**Threshold benefit: Chance of being melanoma-free at 5 years after adjuvant IFNa-2b treatment with severe side effects**	0.70	0.23	0.75	0.54	0.19	0.50	<0.001
**Minimum risk reduction in melanoma recurrence to accept mild-to-moderate side effects for adjuvant IFNa-2b treatment**	0.65	0.22	0.60	0.46	0.19	0.50	0.001

Attitudinal questions showed significant differences in three items: When asked, how they would rate having a bad case of the flu (“fever, headache, nausea, aches and pains”) physicians indicated significantly a lower health state on a scale of 0 to 100 (physicians' mean 38.9 ± 19.6 SD versus patients' mean 46.1 ± 19.1 SD, p=0.004). Patients agreed more strongly with the statement “Having my cancer return after taking a treatment with strong side effects would be better than having my cancer return without taking that treatment” (3.52 ±1.30 versus SD 3.98 ±1.24 SD on average on a scale from 1 to 5, p=0.004). In contrast, physicians more strongly agreed with the statement “There is someone to take care of me, no matter how sick I get.” (1.54 ±0.79 SD versus 1.79 ±0.96 SD, p=0.042).

### Association between utilities and socio-demographics as well as clinical parameters

We found no association of gender or domestic circumstances with utilities or threshold benefits. Having related persons affected by cancer also did not have an impact as reported previously, patients with self-experience with cancer did not have higher utilities (they do not rate the given scenarios more positively because they had already experienced and managed situations of toxicity) but showed higher threshold benefits for the chance of being melanoma-free at 5 years after adjuvant IFNa-2b treatment with mild-to-moderate side effects (60.7% versus 50.7% on average, p=.011).

The same phenomenon could be observed in case of severe side effect. Participants with self-experience of cancer needed a 68.5% chance of 5-year DFS versus 55.3% in participants without this medical history (p=.007). A subgroup analysis could not be performed for the physician cohort due to the low number of physicians with self-experience with cancer (n=5).

### Association between utilities and physicians' professional experience

Both professional experience and frequency of contact with melanoma patients varied strongly between the physicians. A high number of IFN prescriptions was associated with a lower minimum risk reduction for melanoma recurrence demanded by the physicians in context of an IFN therapy with mild-to-moderate side effects (r=-.3, p=.022). In case of severe side effects a lower threshold for the chance of being melanoma-free at 5 years after adjuvant IFNa-2b treatment was needed (r=-.2, p=.003). The proportion of patients undergoing adjuvant IFN therapy with severe side effects was not statistically significant (r=-.25, p=.003). associated with a higher utility for scenario A (no side effects).

## DISCUSSION

The present study evaluates preferences of dermatooncologists in Germany in order to quantify physicians' attitude towards adjuvant IFN therapy. Our physician cohort was younger and consisted of more female subjects as compared to the patient cohort. With a median of 5 years of professional experience, the physician collective was typical for the situation in university hospitals and reflects the current trend in dermatooncology.

In the first part of our trial, we evaluated preferences of German melanoma patients for IFNa-2b toxicity versus recurrence in the adjuvant setting [10]. We found high utilities with median values of 0.99 for IFNa-2b treatment without side effects and mild-to-moderate side effects and abnormal blood test results. Even in case of severe side effects, patients had high utilities. Utilities for melanoma recurrence were considerably lower. High utilities even in case of severe side effects and much lower utilities in case of recurrence suggested that most subjects were willing to accept severe side effects to avoid melanoma recurrence.

The second part adds to this by evaluating the respective preferences of their treating dermatooncologists. In general, we found higher mean and median utilities in physicians (i.e., a more positive rating of the scenarios). It may be that physicians rate disease scenarios more positively because of their knowledge about medical treatment opportunities. As the physician cohort was much younger, this could be an alternative explanation, as younger subjects may anticipate to feel less bothered by side effects.

The median utilities of scenario A to C only marginally differed, while the median utilities for the scenarios of melanoma recurrence (scenario E and F) differed strongly with physicians indicating much higher utilities – maybe because of the medical knowledge about future treatment options. We evaluated that mean threshold benefits and the required minimum risk reduction were lower in physicians. This goes along with a group of 127 metastatic colorectal patients and 150 oncologists, where physicians were willing to tolerate higher toxicity risks than patients [8]. In contrast, in an adjuvant breast cancer cohort, physicians' preferences were the opposite, particularly for modest survival benefits. Physicians were less likely than patients to accept chemotherapy for a small chance of benefit (e.g. 34% of patients vs. 5% of physicians would definitely consider chemotherapy worthwhile for 2 months of benefit) [9]. For a greater benefit, patients' and physicians' choices were more similar (84% of patients vs. 92% of physicians would definitely consider chemotherapy worthwhile for 24 months benefit) [9].

In our study, both groups showed high differences for the chance of a 5-year DFS. For the decision making process, it could important to be aware of these differences. In a discrete choice experiment trial physicians and patients were asked to rate hypothetically how they weigh toxicity versus survival. In total, 200 patients and 226 answered: survival was most important to patients (33%), followed by side effects (29%) and response rate (25%). For oncologists, toxicity was most important (49%), followed by survival (34%) and response rate (12%) [13].

Attitudinal questions showed significant differences in three questions: Physicians seemed to be influenced negatively in a higher extent by physical symptoms than patients. They also disagreed more strongly with the idea that the cancer will come back after a bothering therapy instead of a relapse without this precedent burden of therapy. Physicians were also more strongly convinced that somebody would take care of them in case of a disease. These three questions showed that the physicians of our cohort rated differentially and therefore may be also in a different setting to value treatment outcomes and side effects.

Interestingly, a high proportion of up to 20.4% of physicians and to a lesser extent also patients appreciated even the situation of severe side effects, which also reflects the fact that some individuals are willing to accept even severe side effects. This is important to take into account in the decision making process. The question of concrete expectations toward a cancer treatment should be discussed thoroughly with the patient.

In our physician cohort, a high number of IFN prescriptions was weakly correlated with a lower minimum risk reduction for melanoma recurrence by IFN therapy with mild-to-moderate side effects. Obviously, physicians who prescribe IFN very often seem to appreciate already a smaller treatment benefit. Surprisingly, physicians' utilities did not differ by the proportion of their patients undergoing adjuvant IFN therapy who had severe side effects. Only scenario A was rated more positively by these physicians, we had assumed that physicians more familiar with the toxicity profile of IFN might be more negatively influenced and rate side effects worse than (naïve) patients. Thus, attitudes towards IFN side effects may be triggered more negatively by the fact of being affected themselves than by professional experience.

For a patient-based treatment decision, the individual willingness to accept treatment related toxicity should be evaluated. An inadequate decision making process, on the other hand, can be associated with worse patient-reported health outcomes, worse established quality indicators, and higher healthcare utilization [14].

In conclusion, our study demonstrated that it is important to take into account that different perspectives between patients and physicians are possible and should be regarded with caution.

## PATIENTS AND METHODS

Ten German melanoma centers (9 university hospitals and one community hospital) who were experienced in treating melanoma patients contributed to this non-interventional trial.

In Germany, the treatment of melanoma patients is managed mainly by dermatologists with the subspecialty dermatooncology. 108 physicians, all of them dermatooncologists who were familiar with the treatment of melanoma, were recruited.

The comparator group comprised 130 low-risk melanoma patients, with low-risk being defined as T1a (AJCC 2009 [2]), no sentinel node biopsy performed or significant co-morbidities [10]. The reason for this comparator group was that low-risk melanoma patients had the general experience of a cancer diagnosis but without the concrete conflict to vote for or against IFN in a real-life setting.

### Questionnaires

Physicians and patients completed a standardized paper-based questionnaire. The preferences were measured by the standard gamble method [11] in which patients and physicians rated their preference for a defined health state on a scale from 0 (instant painless death) to 1.0 (perfect health). Using this method, subjects rated two test scenarios and six health state scenarios. Subjects further answered threshold questions to quantify the minimum benefit of 5-year disease-free survival (DSF) they would need to tolerate IFN-related toxicity.

Four of the scenarios presented a range of possible adverse events during IFN therapy: (A) IFN without side effects, (B) IFN with mild-to-moderate side effects, (C) IFN with laboratory abnormalities (liver toxicity and myelotoxicity) requiring dose reduction and causing mild-to-moderate side effects, (D) IFN with severe clinical side effects also requiring dose reduction. Scenario E was melanoma recurrence after IFN therapy with mild-to-moderate side effects and then melanoma death. Finally, scenario F was melanoma recurrence without adjuvant IFN therapy and cancer-death.

### Threshold questions and attitudinal questions

Additionally to the standard gambles that described the subjects' preferences in our trial towards particular conditions, we directly assessed the physicians' preferences for IFN by threshold questions, asking them to choose between a 25% chance of being melanoma-free after five years without any IFN treatment and alternatively IFN with mild-to-moderate side effects. We asked for the minimum chance to stay melanoma-free five years after treatment they would need to accept mild-moderate or severe side effects. In addition, we asked the subjects attitudinal questions to investigate correlations with the utilities.

### Specific items in the physician cohort

The following additional data were collected only for the physicians: frequency of contact with melanoma patients, frequency of IFN prescriptions, professional experience (number of years as a dermatologist), percentage of subjects treated with mild side effects of adjuvant IFN, percentage of subjects treated with severe side effects of adjuvant IFN.

### Statistics

Utilities were calculated according to Kilbridge et al. [12]. Utility for a disease-free health state was defined as U_0_=1.0. The association of utilities with (a) socio-demographics and clinical parameters of physicians and patients, (b) threshold questions, and (c) attitudinal questions were evaluated by Spearman correlations or Mann-Whitney tests.

Differences in socio-demographic data were tested with the help of Chi-square and Mann-Whitney tests, depending on the scaling of the variables.
